# Reviewing migraine-associated pathophysiology and its impact on elevated stroke risk

**DOI:** 10.3389/fneur.2024.1435208

**Published:** 2024-08-01

**Authors:** Vikas Ravi, Sima Osouli Meinagh, Reza Bavarsad Shahripour

**Affiliations:** ^1^Department of Neurosciences, University of California, San Diego, San Diego, CA, United States; ^2^Department of Neurology, School of Medicine, Shahid Beheshti University of Medical Sciences, Tehran, Iran

**Keywords:** migraine disorders, migraine with aura, migraine without aura, ischemic stroke, hemorrhagic stroke

## Abstract

Migraine affects up to 20 percent of the global population and ranks as the second leading cause of disability worldwide. In parallel, ischemic stroke stands as the second leading cause of mortality and the third leading cause of disability worldwide. This review aims to elucidate the intricate relationship between migraine and stroke, highlighting the role of genetic, vascular, and hormonal factors. Epidemiological evidence shows a positive association between migraine, particularly with aura, and ischemic stroke (IS), though the link to hemorrhagic stroke (HS) remains inconclusive. The shared pathophysiology between migraine and stroke includes cortical spreading depression, endothelial dysfunction, and genetic predispositions, such as mutations linked to conditions like CADASIL and MELAS. Genetic studies indicate that common loci may predispose individuals to both migraine and stroke, while biomarkers such as endothelial microparticles and inflammatory cytokines offer insights into the underlying mechanisms. Additionally, hormonal influences, particularly fluctuations in estrogen levels, significantly impact migraine pathogenesis and stroke risk, highlighting the need for tailored interventions for women. The presence of a patent foramen ovale (PFO) in migraineurs further complicates their risk profile, with device closure showing promise in reducing stroke occurrence. Furthermore, white matter lesions (WMLs) are frequently observed in migraine patients, suggesting potential cognitive and stroke risks. This review hopes to summarize the links between migraine and its associated conditions and ischemic stroke, recognizing the profound implications for clinical management strategies for both disorders. Understanding the complex relationship between migraine and ischemic stroke holds the key to navigating treatment options and preventive interventions to enhance overall patient outcomes.

## 1 Introduction

Migraine, a primary headache disorder, is the second leading cause of disability affecting up to 20% of the population globally (1). Stroke is the second cause of mortality and the third leading cause of disability worldwide (2). Previous studies reported a positive association between migraine and ischemic stroke (IS), especially among those less than 45 years old (3). The proportion of migraine-associated strokes in individuals younger than 50 years old is about 25% of all strokes (4, 5). In a prospective cohort study of 63,575 migraineurs and 76,936 individuals without migraine, participants with migraine had a 2.5 times greater risk of developing acute ischemic stroke compared to non-migraineurs (6). Epidemiological studies, have shown that a migraine attack can lead to an ischemic stroke in about 0.1–0.2% of cases, with the risk increasing to up to 1% in younger patients (7–9). The correlation between migraine and stroke can be delineated across several dimensions. Firstly, migraine and stroke share a common pathophysiology, suggesting a foundational link that explains their concurrent presence. Secondly, there is a bidirectional relationship where migraine can predispose individuals to stroke, and conversely, individuals may experience migraines for the first time following a stroke. Lastly, migraine’s ability to mimic the symptoms of a stroke—often referred to as “stroke chameleon”—can complicate diagnosis and treatment, highlighting the complexity of these intersecting conditions (10).

Migraine-associated stroke is separated into “migraine-related stroke” and “migrainous infarction.” Migraine related stroke is a interictal stroke occur in a patient with migraine headache history (2). On the other hand, according to the International Classification of Headache Disorders, 3rd Edition (ICHD-3), migrainous infarction is diagnosed based on specific criteria: a history of migraine with aura (MA), acute aura symptoms that persist for more than 1 h and are similar to previous aura episodes, and imaging findings that show defects correlating with the aura symptoms. However, this diagnostic approach has limitations, particularly in identifying migrainous infarction in patients who do not experience aura or in cases where the symptoms do not align with previous aura attacks. This can lead to challenges in accurately diagnosing migrainous infarction in these subsets of patients (11, 12). Migraine triggers lead to pain by initiating a series of physiological responses, including inflammation, increased cortical excitability, and arterial dilation. These processes are key in the development of migraine symptoms. It can be with aura or without aura (12, 13).

**Figure 1:**
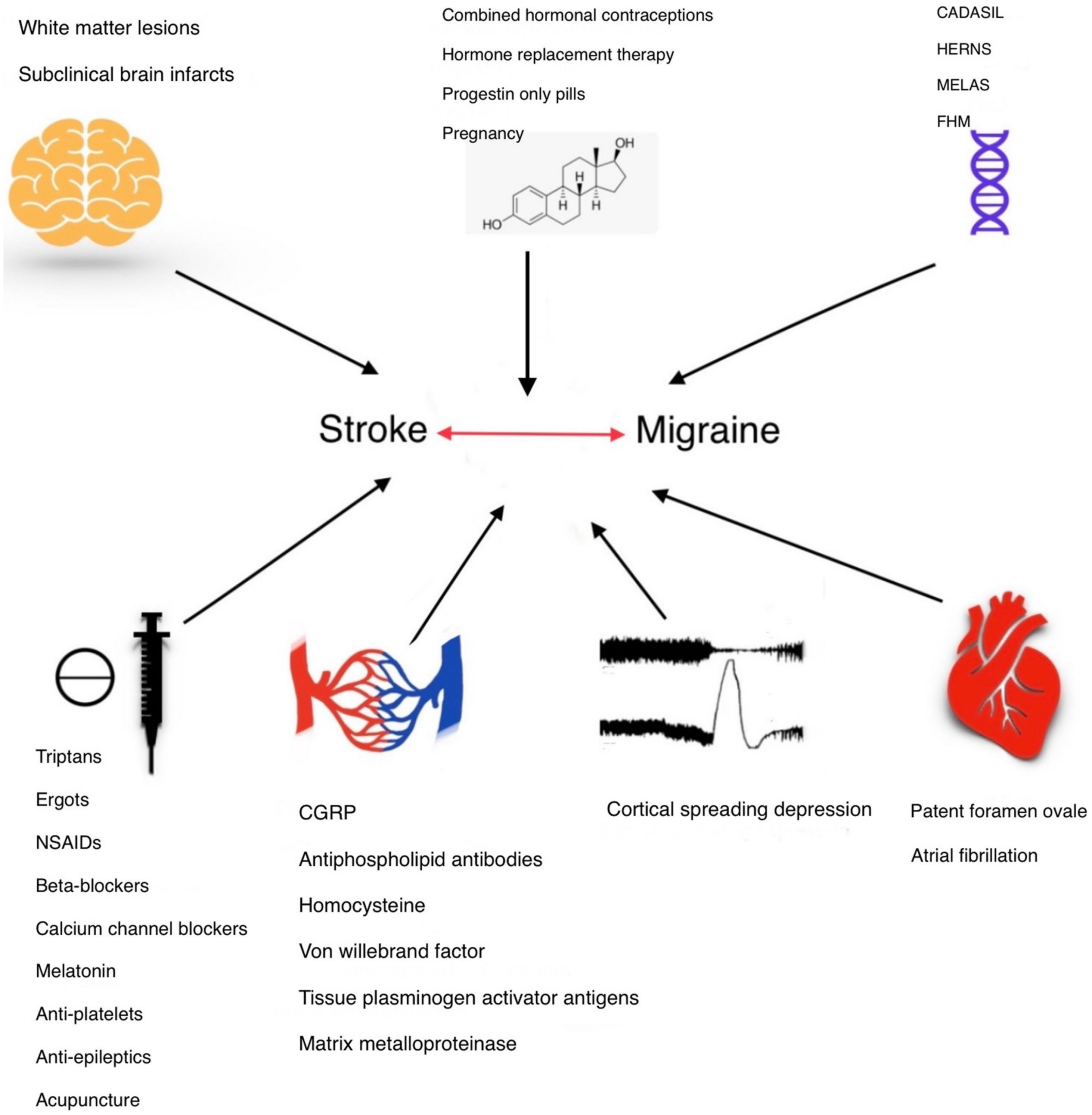
The association between migraine and stroke can be defined in different aspects. Common genetic locus and mutations in syndromes like autosomal dominant arteriopathy with subcortical infarcts and leukoencephalopathy (CADASIL), hereditary epitheliopathy, retinopathy, nephropathy, and stroke (HERNS), mitochondrial encephalomyopathy, lactic acidosis, stroke-like symptoms (MELAS), and Familial hemiplegic migraine (FHM) express this connection. Estrogen level changes which can be studied with hormonal therapies, during pregnancy and menstrual periods, can affect both migraine and stroke. White matter changes on the other hand can help physicians and researchers to define their connection and estimate stroke risk in migraineurs. Antimigraine medications and post-stroke therapies could influence migraine and stroke links. Endothelial dysfunction proposes a valuable understanding of the underlying pathophysiology. Understanding the role of cortical spreading depression in migraine and stroke could improve our insight into this association. Patent foramen ovale (PFO) serves as a potential comorbid condition of stroke and migraine, especially migraine with aura.

In this review, we aim to conduct a comprehensive literature review on the correlation between migraine and stroke, considering all predisposing factors and potential risk factors. Our goal is to systematically analyze existing studies to better understand how various elements linked to migraine may influence the risk of stroke and to identify gaps in the current research landscape for future investigation. Figure 1, summarizes the topics we have discussed in this review.

## 2 Migraine in ischemic and hemorrhagic stroke

The risk of IS in migraineurs with less than one aura attack per month and more than one attack per week is twofold and fourfold, respectively (5). However, the relationship between migraines and hemorrhagic stroke (HS) remains inconclusive, suggesting that while migraines are a clear risk factor for ischemic events, their impact on HS needs further exploration (14).

Sacco et al. in a meta-analysis of eight studies, which included 1,600 cases of HS, found an increased risk of HS among female migraine sufferers, particularly those under the age of 45. However, this analysis reported no significant association between MA and HS, highlighting the complexity and variability of how migraines interact with different types of stroke risks (15). Hu et al. conducted a comprehensive meta-analysis involving 11 studies and over 2.2 million participants. They found a positive correlation between migraine and an increased risk of total IS, but no significant correlation with HS. Further subgroup analysis revealed that MA is at a significantly higher risk for both IS and HS, while those without aura did not show the same increased risk. This suggests that the presence of aura in migraine sufferers may be a key factor in their heightened vulnerability to strokes (16). This variability underscores the need for more targeted research to understand the specific risk factors associated with migraines and HSs.

Ta-Thi et al. in a recent meta-analysis, found a positive correlation between migraine and total stroke, both IS and HS, when analyzing data using a fixed-effect model. However, the correlation was only significant between migraine and total stroke when using a random-effects model. Additionally, subgroup analysis showed no significant association between migraine and specific types of strokes, such as IS and HS (17).

IS in patients with migraine can be classified into three distinct categories: First, IS that coincides with migraine but is caused by other factors; second, IS where the symptoms closely resemble those of MA, but the underlying causes are unrelated to migraine; and third, migraine infarction, where the IS is directly linked to a migraine episode, specifically occurring during or shortly after a MA (18). There is ongoing debate regarding whether only MA contributes to the risk of IS, or if migraine without aura (MO) can also increase the likelihood of stroke. This controversy stems from differing findings in research studies, highlighting the need for further investigation into the specific impacts of both types of migraine on stroke risk (19). Adnyana et al. in a recent meta-analysis indicated that both MA and MO could be defined as risk factors for IS, reporting a 1.1-fold increase in risk for MO, a 1.4-fold increase for MA, and a combined effect of a 1.2-fold increase in stroke risk (20).

The hypothesized mechanisms for the increased risk of IS in individuals with migraine, particularly those with aura, include cortical spreading depression (CSD), a higher incidence of patent foramen ovale (PFO), and a greater prevalence of systemic lupus erythematosus and antiphospholipid syndrome. These factors are believed to contribute to the heightened vulnerability to IS observed in migraineurs (21).

## 3 Migraine-associated pathophysiology contributing to elevated stroke risk

### 3.1 Genetic predisposition

In a Mendelian randomization study, Wang et al. found that a genetic predisposition to migraine—regardless of specific migraine subtypes—was associated with poorer outcomes on the modified Rankin Scale (mRS) in acute stroke patients after 3 months. This finding suggests that genetic factors linked to migraine may influence the severity of stroke recovery, highlighting the potential impact of migraine-related genetics on long-term stroke outcomes (22).

Cervical artery dissection, involving the vertebral and carotid arteries, is a prominent cause of IS in young and middle-aged adults (23). Sun et al. recently conducted a meta-analysis and found that individuals with migraine have a 1.74-fold increased odds of experiencing cervical artery dissection. Notably, this association was specifically significant for those suffering from WO, and the risk was consistent across both genders (24). Several genetic studies have identified common genes that predispose individuals to both cervical artery dissection and migraine. Notable among these are *AMAMTSL4/ECM1*, *PLCE1*, and *MRVI1*, which are located in the same genomic loci (25). Additionally, it has been observed that individuals with migraines tend to have higher concentrations of elastase, an enzyme that degrades the extracellular matrix. This enzymatic activity weakens the structural integrity of vessel walls, making them more susceptible to dissection. These findings provide a genetic and biochemical link between migraine and an increased risk of cervical artery dissection (26).

Gollion et al. have reported that young patients with migraine are less prone to the large vessel atherosclerosis subtype of IS compared to non-migraineurs (27). Genetic research supports this finding, showing a lower prevalence of alleles related to coronary artery disease (CAD) in migraineurs, such as *PHACTR1* gene polymorphism, which has opposite effects on migraine and CAD (28). Polygenic scores and genome-wide association studies (GWAS) further reveal that the genetic overlap is more pronounced between MO and IS than between MA and IS (29). This genetic association is particularly significant for large-artery stroke (LAS) and cardioembolic stroke (CE), rather than for small-vessel disease. These findings contrast with earlier reports highlighting a stronger relationship between MA and IS than MO and IS, hypothesized to be due to rare genetic loci associated with MA not detectable in GWAS (30).

The deletion of the angiotensin-converting enzyme (ACE) gene is linked to a hypercoagulable state and vasoconstriction, which can lead to stroke. Additionally, this genetic alteration is associated with an increased frequency of migraine attacks. This finding highlights the role of genetic factors in influencing both vascular health and the severity and occurrence of migraines, illustrating the complex genetic interactions that affect these conditions (31). Another genetic polymorphism that can increase the risk of IS in migraineurs is a mutation in methylene tetrahydrofolate reductase (MTHFR) which encodes enzymes responsible for folate and homocysteine metabolism (31).

The relationship between migraine and stroke can also be defined in some syndromes like cerebral autosomal dominant arteriopathy with subcortical infarcts and leukoencephalopathy (CADASIL), hereditary epitheliopathy, retinopathy, nephropathy, and stroke (HERNS), mitochondrial encephalomyopathy, lactic acidosis, stroke-like symptoms (MELAS), and Familial hemiplegic migraine (FHM). Each of these conditions features a combination of migraine and stroke symptoms, underscoring the genetic and pathological connections between these two neurological disorders (32).

In CADASIL, which is associated with mutations in the *NOTCH3* gene, fibrosis of the perforating cerebral arteries leads to subcortical infarcts and leukoencephalopathy. Typically, the initial manifestation in affected individuals is MA, which is often followed by IS later in life. As these stroke events occur, the frequency of migraine attacks tends to decrease or even cease altogether. Preclinical studies have shown that mice with similar mutations display increased vulnerability to spreading depression (SD) (33). The aura experienced in CADASIL is usually more complicated and long-lasting and tends to occur at an older age compared to other migraine sufferers (18). MRI scans of patients invariably show abnormal results, including bilateral infarcts in the deep white matter and periventricular regions (34). Due to the risk of vasoconstriction, the use of triptans is contraindicated in individuals with CADASIL (4).

HERNS is an autosomal dominant syndrome, that manifests with migraine-like headache, psychiatric disturbance, dysarthria, hemiparesis, and apraxia with subcortical contrast-enhanced lesions in imaging (35).

In MELAS, caused by mutations in the *MT-TL1* gene, early symptoms typically appear in childhood and include tonic–clonic seizures and migraines with abdominal symptoms, followed by progressive neurological deficits such as hearing loss. Diagnostic features include calcifications in the basal ganglia visible on neuroimaging, and elevated lactate levels in both blood and cerebral spinal fluid, which are key indicators of this mitochondrial disorder (18).

Familial hemiplegic migraine (FHM) is a rare autosomal dominant neurological disorder linked to mutations in several genes, including *CACNA1A*, *ATP1A2*, *SCN1A*, and *PRRT2*. It is characterized by reversible motor weakness that often leads to its misdiagnosis as a stroke or transient ischemic attack (TIA). Despite its stroke-like symptoms, FHM itself can predispose patients to genuine ischemic events, underscoring the need for accurate diagnosis and understanding of its distinct genetic basis and clinical manifestations (34).

This overview accentuates the critical need for specialized therapeutic strategies and preventive actions, improving treatment plans for individuals dealing with both migraine and stroke.

### 3.2 Endothelial dysfunction

Cerebral blood flow autoregulation is a complex mechanism vital for sustaining stable brain function. It encompasses both static and dynamic characteristics, crucial for maintaining basal vessel tone and promptly responding to sudden alterations (36). To investigate the impact of migraine on cerebral vascular reactivity (CVR), researchers have employed various investigative approaches, which include hypercapnia, hypocapnia, and chemical infusions to stimulate vascular responses, alongside visual stimuli, to provoke neuronal reactions (37). Results indicate that visual stimuli can increase CVR between migraine attacks compared to controls. However, the effects of vascular stimuli on CVR have shown inconsistent results, highlighting the dominance of central neuronal mechanisms over peripheral vascular mechanisms in influencing migraine attack modulation (37–39).

Trigeminal ganglion disinhibition results in the secretion of calcitonin gene-related peptide (CGRP), precipitating cerebral vasodilation. CGRP serves as a crucial link between the neuronal and vascular components of migraine headaches (3). Interestingly, endogenous CGRP also exerts protective effects, mitigating the progression of infarct size and ameliorating cerebral vasospasm following subarachnoid hemorrhage (33). This vasodilatory effect of CGRP could potentially decrease the occurrence of IS following a migraine attack (33). However, contrary to what might be expected given these protective effects, Fanvoni et al. reported that CGRP-targeted preventive treatments do not increase cardiovascular events in migraineurs, suggesting that while CGRP has beneficial effects on vascular dynamics, its modulation does not necessarily lead to adverse cardiovascular outcomes (40).

Neoangiogenesis, the process of forming new blood vessels, emerges as a potential mechanism for reducing disability post-stroke. Notably, migraineurs exhibit lower levels of endothelial progenitor cells compared to non-migraineurs, potentially predisposing them to greater disability post-stroke (10). However, findings from a prospective cohort study of women migraineurs over the age of 45 showed a contrasting outcome. Rist et al. reported that the MA group had a two-fold higher likelihood of achieving a mRS score of 0 or 1, indicating no or minimal disability, compared to the control group (41). These findings diverge from those of a recent Mendelian randomization study, suggesting that patients genetically predisposed to migraine, irrespective of subtype, experience worse mRS outcomes 3 months post-stroke (22). This conundrum highlights the complex interplay among migraine, genetic predisposition, and post-stroke recovery outcomes.

Previous studies have identified elevated concentrations of procoagulants such as antiphospholipid antibodies, homocysteine, von Willebrand factor, tissue plasminogen activator antigens, and matrix metalloproteinase (MMP) in migraineurs, supporting the link between migraine and stroke risk (5, 42–44). MMPs, in particular, are implicated in hemorrhagic transformation, brain swelling, and increased infarct size in IS, and their inhibition is considered a therapeutic avenue in stroke management (45, 46). Notably, a recent study has shown that MMP activity is heightened in both MA and MO, potentially amplifying both the risk and severity of stroke occurrences (2, 47, 48).

### 3.3 Cortical spreading depolarization

Cortical spreading depolarization (CSD), a slow-moving wave of depolarization coursing through brain cells at a rate of roughly 3 mm/min, is a fundamental mechanism implicated in various neurological disorders, including IS, intracranial hemorrhage, subarachnoid hemorrhage, traumatic brain injury, transient global amnesia, and notably, MA (34, 49). This phenomenon highlights the interconnectedness of these diverse conditions, suggesting shared pathological processes ripe for targeted treatment strategies.

During a migraine, CSD triggers a transient surge in brain tissue blood flow lasting 1–2 min, increasing energy, oxygen, and glucose consumption. This is subsequently followed by a period of hypoperfusion lasting 1–2 h. Repeated CSD occurrences from migraine attacks may diminish neuronal resilience overall and accelerate ischemia onset (10). Inflammatory cytokines such as endothelial microparticles (EMP), endothelial progenitor cells (EPCs), high-sensitivity C-reactive protein (hs-CRP), and endothelin-1 serve as biomarkers of endothelial dysfunction during CSD in migraine, shedding light on vascular and inflammatory alterations exacerbating migraine episodes (43).

EMP, released in response to inflammatory cytokines, plays a critical role in attracting leukocytes to sites of inflammation and suppressing nitric oxide (NO) synthesis, thereby initiating thrombosis (43). The level of endothelin-1, a vasoconstrictive biomarker produced by endothelial cells, neurons, and glial cells, has been found to increase during the ictal phase of migraine headaches, predisposing patients to stroke through vasoconstriction (2). Endothelin-1 also influences CSD, highlighting its significant role in the pathophysiology of migraine and stroke risk (2).

Preclinical studies indicate that CSD episodes markedly increase cerebral metabolic rate and oxygen consumption while concurrently diminishing cerebral blood flow, seen particularly in migraineurs (33, 50). Moreover, elevated oxidative stress markers are observed in migraineurs compared to controls, suggesting intensified and prolonged cerebral responses to CSD, potentially contributing to the disorder’s chronicity and risk of further complications (10).

The link between ischemic stroke and migraine is also seen when analyzing the mechanisms of Na, K-ATPase-dependent cellular signaling. Increased sensitization of vascular muscle cells post-stroke leads to vasoconstriction and subsequent worsening of stroke symptoms. By inhibiting cSrc kinase, a key component of the Na, K-ATPase signaling pathway, it is possible to reduce this post-stroke vasoconstriction. This reduction is achieved by lowering the cells’ sensitivity to calcium and decreasing the production of reactive oxygen species, particularly noted following reperfusion therapy for IS. These observations suggest potential therapeutic avenues for alleviating the vascular complications observed not only during stroke recovery but also in migraine (51). Blood flow reduction can trigger CSD, a shared pathophysiological process in both MA and IS. This reduction in blood flow stimulates the secretion of ouabain, a cardiotonic steroid, which induces vasoconstriction to help maintain blood pressure. Ouabain inhibits Na, K-ATPase, leading to an increase in extracellular potassium (K^+^), thereby precipitating membrane action depolarization, and propagating depolarization waves. Such mechanisms underscore the complex interplay between vascular changes and neurological events in conditions like migraine and stroke, highlighting potential targets for therapeutic intervention (51).

In summary, understanding CSD’s role in these conditions is vital for developing targeted interventions to mitigate stroke risk in migraine sufferers.

### 3.4 Patent foramen ovale

Fetal circulation relies on a right-to-left shunt in the atria, facilitated by a physiological foramen, to function properly. Postnatally, the increase in pulmonary blood flow and pressure in the left atrium typically prompts the closure of this foramen through fusion of the septum primum and septum secundum (52). However, the patent foramen ovale (PFO) may persist beyond infancy into adulthood, remaining open in approximately 25% of adults (53, 54).

The prevalence of PFO varies considerably depending on various conditions. In cases of stroke, PFO prevalence ranges from 23.5 to 61.1%. In cases of MA, the prevalence of PFO ranges from 19 to 77.9%, whereas for MO, it ranges from 11 to 34.1% (55). This variability highlights the potential link between PFO and these neurological conditions, particularly stroke and MA.

One of the hypothesized mechanisms of IS in patients with patent foramen ovale (PFO) involves a right-to-left shunt, which allows for the passage of emboli from the right to the left side of the heart. This condition, coupled with predisposing factors such as hemodynamic instability and elevated platelet function in the left atrium, can facilitate the formation and migration of emboli to the brain, potentially leading to IS. This pathway underscores the critical interplay between structural heart anomalies and vascular risk factors in stroke pathogenesis among PFO patients. Animal studies indicate that the size of microemboli reaching the brain significantly influences outcomes; larger microemboli can cause brain tissue necrosis and blood–brain barrier damage, while smaller ones may induce cortical suppression diffusion, characteristic of MA (56, 57).

A meta-analysis by Rengifo-Moreno et al. focusing on patients with coexistent stroke or TIA and PFO demonstrated that device closure of the PFO significantly reduced the risk of subsequent stroke or TIA when compared to medical therapy alone, with a pooled hazard ratio (HR) of 0.59 and a 95% confidence interval (CI) of 0.36–0.97 (*p* = 0.04) (58). Further research by Schwedt et al. indicated that approximately 80% of patients with cryptogenic stroke and a history of migraine exhibited a right-to-left shunt due to PFO (59). Similarly, Wolf et al. reported that 64.7% of migraine infarctions were associated with the presence of a PFO, underscoring the significant link between PFO and neurological events like stroke and MA (9).

Another hypothesis for migraine occurrence in PFO patients involves altered activity of serotonin (5-HT) receptors due to increased platelet activity and decreased lung perfusion, possibly stemming from heightened oxidative stress observed in PFO cases (60, 61).

Nevertheless, randomized control trials (RCT) reported no significant benefit from PFO closure in migraineurs (62–64). Tobis et al. reported participants in the PFO closure group experienced a greater reduction in headache days and higher rate of complete migraine remission for 1 year in comparison to the control group. However, there was no significant difference in the 50% reduction of migraine attacks in the two groups (64). In another RCT of 107 participants, PFO closure did not reduce the frequency of monthly migraine attacks and led to five adverse events (63).

Notably, clopidogrel, an antiplatelet drug commonly used for secondary IS prevention, has been observed to reduce migraine attack frequency in individuals with an open PFO, suggesting potential therapeutic benefits beyond stroke prevention in managing PFO-associated migraines (65).

### 3.5 Atrial fibrillation

Atrial fibrillation (AF) serves as another comorbidity in both IS and MA. Atrial fibrillation is one of the underlying predisposing factors for IS, however its association with MA is bidirectional. Autonomic dysfunction during a migraine attack can initiate AF and on the other hand, AF can induce CSD with an emboli (56, 66).

In a cohort study of 11,939 participants and 20 years of follow-up, migraine with visual aura was related to a greater risk of AF in comparison to migraineurs without visual aura and nonmigraineurs (67). A Danish population-based study, revealed a positive association between migraine and AF and atrial flutter (OR 1.25, 95% CI 1.16–1.36), which was more significant in MA patients (68).

De Giuli et al. reported, that migraineurs within a stroke patient population had a lower incidence of AF compared to non-migraineurs (69). Conversely, Gollion et al. found no significant differences in the causes of stroke between patients with and without migraine, but AF was more common in those with MA (10.34%) than non-migraineurs (2.2%, *p* = 0.011, 70). These conflicting results can be attributed to the differences in study populations (70).

### 3.6 Hormone-mediated risks and gender disparities

Estrogen-associated migraines are triggered by fluctuations in estrogen levels from both endogenous and exogenous sources, such as natural hormonal shifts during the menstrual cycle’s luteal phase, or from external factors like combined hormonal contraceptives (CHC) or hormone replacement therapy (HRT) respectively (71, 72).

MA and MO exhibit distinct responses to estrogen fluctuations. Estrogen withdrawal typically triggers MO, leading to a higher prevalence of headaches during the 2 days preceding menstruation and the first 3 days of the menstrual period. Conversely, MA headaches often arise during periods of elevated estrogen, such as in pregnancy or while using CHC or HRT (72–76).

CHC independently heightens the risk of thrombotic stroke (77). When combined with MA, this risk escalates significantly, necessitating caution when prescribing CHC to such individuals (77, 78). Studies have shown that CHC consumption can increase stroke risk up to sevenfold in women with MA (79). Champaloux et al. reported an odds ratio (OR) of 6.1 for stroke in women with MA and an OR of 1.77 in women with MO who had used CHC within 3 months before a stroke event (77). To mitigate the increased risk of stroke in women with MA, alternative contraceptive methods may be warranted (77).

The reduction in estrogen levels during the luteal phase of the menstrual cycle increases vascular permeability, which in turn elevates the levels of inflammatory mediators, thereby increasing migraine susceptibility. The decline in estrogen can additionally lead to a decrease in serotonin levels and a suppression of opioid tone within the CNS, which contribute to the onset of migraine symptoms, as both serotonin and opioid systems play crucial roles in pain regulation and mood stabilization (21). Conversely, high estrogen levels have been associated with an increase in the frequency of aura symptoms. Interestingly, low-dose oral CHC containing 10–15 μg of ethinyl estradiol appears to inhibit ovulation, decrease aura frequency, and reduce stroke risk (80). Progestin-only pills (POP) do not increase the risk of stroke but have been reported to decrease the frequency of migraine attacks and reduce the need for analgesics. However, consistent POP consumption is crucial for contraceptive efficacy due to its short half-life (81).

Variability in stroke risk findings associated with CHC can be attributed to differing estrogen doses used across studies. Historically, before 2002, many studies indicated a significantly increased risk of stroke associated with CHC formulations containing at least 50 μg of estrogen (81). However, more recent studies have adjusted the estrogen dosage and observed differing results. For instance, Lidegaard et al. in a case-control study reported no increased risk of stroke with CHC containing 20 μg of estrogen, while an increased risk was noted in women consuming pills with 50 μg of estrogen (82). Additionally, a large study in the United States involving 3.6 million women, along with another pooled analysis, confirmed that there is no increased risk of stroke among users of low-dose CHC (83). These findings emphasize the importance of estrogen dosage in evaluating cardiovascular risks (81).

Previous studies have observed that migraine headaches can persist in postmenopausal women undergoing hormonal therapy. However, these episodes tend to occur less frequently with continuous transdermal estradiol as compared to conjugated equine estrogens, supporting a preference for transdermal patches in HRT for postmenopausal women with a migraine history (84). Canonico et al. reported that the OR for IS was 1.58 (95% CI 1.01–2.49) in users of oral estrogen and 0.83 (95% CI 0.56–1.24) in users of transdermal estradiol patches for postmenopausal hormone therapy. These findings reinforce the recommendation against oral estrogens in women with migraines, highlighting the lower associated risk with transdermal estrogen administration (85).

During pregnancy, the stability of estrogen levels typically results in a reduction in migraine occurrences (86). However, despite this decrease, pregnant migraineurs still face an elevated risk of IS compared to the general population, illustrating the complexities between hormonal shifts and stroke susceptibility during pregnancy (87). The stroke risk during pregnancy surpasses that of individuals using CHC alone and those experiencing migraines without pregnancy (88, 89). Consequently, considering alternative contraceptive methods other than CHC, such as POP or non-hormonal options, may be prudent to potentially mitigate stroke risk (89).

The European Headache Federation and the European Society of Contraception and Reproductive Health have classified the evidence regarding the safety of oral, transdermal, and vaginal forms of CHC in women with MA as C-level. Due to associated risks, they advocate for the exploration of non-hormonal methods or progestogen-only contraceptives as safer alternatives for these individuals. This recommendation aims to minimize the potential occurrence of stroke and other vascular events in migraineurs sensitive to estrogen-related complications (90).

The lifetime prevalence of migraine is significantly higher in women at 43%, compared to 18% in men, with the most common type being MO, specifically menstrual migraine (91–93). Additionally, the stroke risk for women during their menopausal years is approximately 20% in the United States (94). Notably, younger women under 45 who smoke and use oral contraceptives exhibit the strongest correlation between migraine and IS compared to men (95). Research indicates that the risk of IS in female migraineurs significantly increases when combined with other risk factors, with the risk multiplying 3–9 fold with smoking, 4–8 fold with oral contraceptive use, and up to 10 fold when both risk factors are present (96). This data underscores the critical consideration of lifestyle and hormonal factors in migraine management to mitigate associated risks, particularly in women.

In summary, for women with MA, the preferred contraceptive method involves non-estrogen-containing options unless contraception serves other therapeutic purposes. In cases where additional health concerns such as acne treatment or resistant menstrual-related headaches are present, estrogen-containing contraceptives may be considered. This approach ensures that individual health needs and risks are addressed while minimizing potential complications associated with estrogen in migraineurs (81). Table 1 summarizes the results of case-control studies that have investigated the association between hormonal therapies and stroke.

**Table 1:** Summary of main case-control studies has investigated hormonal therapy on stroke and migraine.

Pub year (ref)	Study type	Population	Main goal	Drug type	Dosage	Results
1996 (80)	Hospital-based, case-control study	697 cases 1962 age-matched hospital controls Aged 20–44 years	The risk of ischemic stroke in association with current use of COCs	COC	Low dose: <50 μg High dose: ≤50 μg	OR for stroke (95% CI): High dose: 5.30, (2.56–11.0) Low dose: 1.53 (0.71–3.31)
1996 (83)	Population-based, case-control study	295 case 295 controls Aged 15–44 years	The relation of stroke to the use of oral contraceptives	OC	High dose is rare in United States	OR for ischemic stroke (95% CI): 1.18 (0.54–2.59) OR for hemorrhagic stroke (95% CI): 1.14 (0.60–2.16)
2002 (82)	Case–control study	626 cases 4,054 controls Aged 15–44 years	The influence of OCs on the risk of CTA	OCs and progestin-only pills	OCs with 50, 30–40, and 20 μg ethinyl estradiol	OR for CTA (95% CI): 50 μg: 4.5 (2.6–7.7) 30–40 μg: 1.6 (1.3–2.0) 20 μg: 1.7 (1.0–3.1) Progestin-only pills: 1.0 (0.3–3.0)
2016 (85)	Nested case-control study	3,144 hospitalized ischemic stroke 12,158 controls Aged 51–62 years	The influence of estrogen consumption rout and dose in ischemic stroke	OCs and transdermal estrogen	Oral estrogens and transdermal estrogens, respectively, low: ≤1 mg/d and <50 μg/d intermediate: 1.5 mg/d and 50 μg/d high: ≥2 mg/d of and >50 μg/d	Adjusted OR for ischemic stroke (95% CI): Oral estrogens: 1.58 (1.01–2.49) (significantly high in low, intermediate, and high dose) Transdermal estrogens: 0.83 (0.56–1.24) (not significant in low, intermediate, and high dose) Norpregnancs: 2.25 (1.05–4.81) no association of IS with use of progesterone, pregnanes, and nortestosterones
2017 (77)	Nested case-control study	1,884 cases 7,536 controls Females ages 15–49 years with first-ever stroke	The incidence of stroke to examine the association among CHC use, migraine type (MA, MO), and ischemic stroke	COC/combined hormonal patch/combined hormonal ring	NM	Cumulative incidence of 11 strokes/100,000 females OR for ischemic stroke (95% CI): MA + CHC: 6.1 (3.1–12.1) MA, no CHC: 2.7 (1.9–3.7) MO + CHC: 1.77 (1.1–2.9) MO, no CHC: 2.2 (1.9–2.7)
2023 (97)	Case-control	127 cases 650 controls Female aged 18–55	To clarify how estrogen dose impact ischemic stroke risks	CHC	Ethinyl estradiol dose: ≥30 vs. <30 μg	OR for ischemic stroke (95% CI)/*p*-value ≥30-μg EE doses compared to <30-μg dose: 1.52 (1.02, 2.26)/0.040

COC, combined oral contraception; OR, odds ratio; CI, confidence interval; OC, oral contraception; CTA, cerebral thromboembolic attacks; CHC, combined hormonal contraceptive; MA, migraine with aura; MO, migraine without aura; NM, not mentioned.

### 3.7 White matter lesions and subclinical brain infarcts

White matter lesions (WML) and subclinical brain infarcts (SBI) represent potential precursors to clinical strokes, cognitive decline, functional impairments, and even dementia (98).

**Figure 2:**
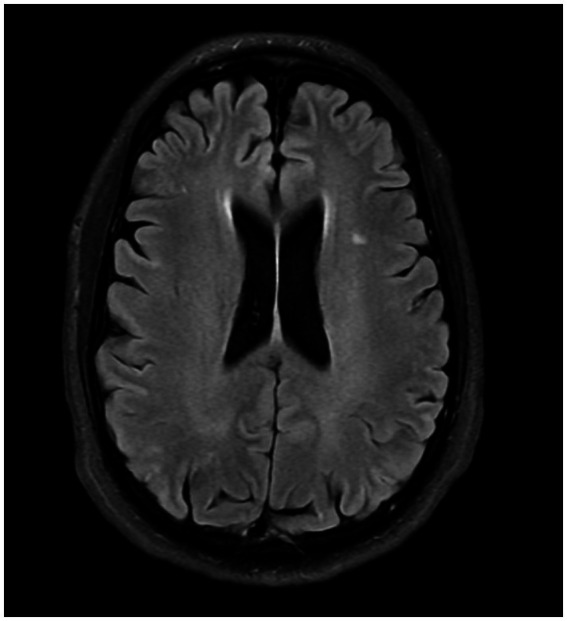
Subclinical brain infarct, FLAIR sequences of MRI.

**Figure 3:**
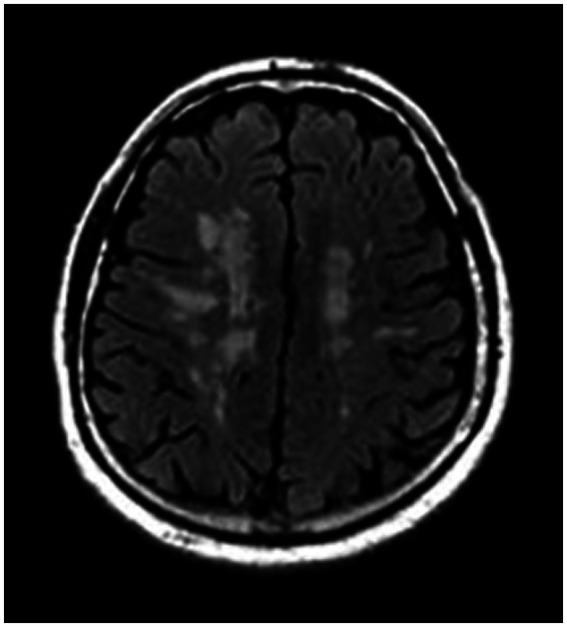
White matter lesion in a patient with chronic migraine headache, FLAIR sequences of MRI.

While WMLs are not exclusive to individuals with migraines, studies have reported that 4–59% of migraine sufferers exhibit WMLs, a rate notably higher than that observed in control groups. This suggests an association between migraine and these brain abnormalities, necessitating further exploration of how migraines may contribute to or exacerbate these conditions (34, 99). Although white matter lesions (WMLs) are reported to be more frequent in migraine sufferers than in the general population, they should be differentiated from silent brain infarcts (SBIs) to accurately reassess stroke risk. Figure 2 demonstrates SBIs, while Figure 3 depicts WMLs related to migraines.

Notably, Kruit et al. demonstrated that migraine patients exhibit a higher incidence of SBI in the posterior circulation territory, particularly pronounced in those with higher migraine attack frequencies or aura experiences (100). Additionally, an increased prevalence of deep WML was observed among migraineurs, particularly women, irrespective of aura status (100). However, the association between periventricular WMLs and migraine was not significant. Longitudinal research over a 9-year follow-up period indicated a rise in WMLs among women with migraines, although the increase in SBIs was not significant (101).

Kurth et al., in a study involving 780 participants with a higher mean age, found a notably higher prevalence of deep WMLs and brain infarcts in individuals with MA (102). Conversely, Monteith et al. reported a higher risk of SBI but not WML among migraineurs (103). However, a Danish twin-based study by Gaist et al. found no significantly higher incidence of SBI or WMH in women with migraine compared to controls (104).

Endothelial NO synthase, which converts L-arginine into NO, facilitates vasodilation and can prevent ischemic manifestations. Asymmetric dimethylarginine (ADMA), and symmetric dimethylarginine (SDMA), which are methylated derivatives of L-arginine, inhibit NO synthase and are linked to cardiovascular morbidity and mortality (105). Erdelyi-Botor et al. found higher concentrations of ADMA and SDMA in migraineurs with WML, with older patients and those with a higher lifetime number of headache attacks exhibiting an increased number of lesions (105).

A recent meta-analysis by Espanol et al. found no significant link between migraine and SBI in population-based studies. However, a broader analysis incorporating both population and clinical-based studies revealed a positive correlation between MA and both SBI and subclinical cerebellar infarcts, indicating potential study type influences on results (106). Additionally, another meta-analysis by Bashir et al. identified MA as a risk factor for WML, while MO showed no such association (99).

These findings underscore the intricate relationship between migraine and structural brain changes, highlighting the need for continued monitoring and research into the long-term neurological impacts of migraine.

## 4 Stroke mimics and stroke chameleons

Within the realm of stroke misdiagnosis, two distinct phenomena emerge: the “stroke mimics,” or false positives, and the “stroke chameleons,” or false negatives (107). Migraine, capable of manifesting as either mimics or chameleons of stroke, stands as the third most common cause of erroneous administration of thrombolytic therapy (108). Terrin et al. conducted a systematic review of articles from 1995 and found that MA accounted for more than 1% of stroke unit emergency evaluations and over 1.5% of thrombolytic treatments. Furthermore, a staggering 18% of incorrect thrombolytic therapies were administered to patients experiencing migraine attacks (108). This underscores the need for careful assessment to discern between migraine manifestations and genuine stroke symptoms.

Aura, characterized by focal neurological deficits sometimes without accompanying headache, is the most common reason migraines mimic stroke (107, 109). Conversely, MO is frequently a major contributor to cases classified as stroke chameleons—situations where stroke symptoms are present but the cause is not a stroke. Additionally, hemiplegic migraine and migraine with unilateral motor symptoms (MUMS), both characterized by motor symptoms, can easily be misdiagnosed as stroke, demonstrating the challenges and potential risks of diagnosing neurological conditions based solely on symptom presentation (107, 110).

In emergency settings, accurately diagnosing IS is crucial to initiate the appropriate therapy and prevent the deterioration of the patient’s condition. To facilitate rapid diagnosis of stroke mimics, two scoring systems have been established:

1. **FABS Score**: This score includes six items—absence of facial droop, no history of atrial fibrillation, age under 50 years, systolic blood pressure below 150 mmHg at presentation, history of seizures, and presence of isolated sensory symptoms without weakness. A FABS score of ≥3 has been shown to have a sensitivity of 91% and specificity of 90% in diagnosing stroke mimics (111).
2. **TeleStroke Mimic-Score (TM-Score)**: This method calculates the likelihood of stroke mimics based on several factors including age, history of atrial fibrillation, history of hypertension, history of seizure, presence of facial weakness, and an NIHSS (National Institutes of Health Stroke Scale) score greater than 14 (112).

These tools are designed to help healthcare providers quickly differentiate between actual strokes and stroke mimics in emergency scenarios, ensuring that patients receive the most appropriate and timely treatment.

Previous studies have noted a higher incidence of TIA in patients with a history of MA. Distinguishing between MA and TIA, particularly in older patients, can be challenging for practitioners. One critical distinguishing feature is the onset of symptoms, which tends to occur more rapidly in TIA compared to MA (42). Fogang et al. identified that older age, male gender, and a history of stroke, hypertension, or dyslipidemia were prevalent risk factors for TIA in patients presenting with transient neurologic deficits (113). Given that TIA is a risk factor for subsequent ischemic attacks, correctly identifying whether a patient has experienced a TIA rather than a migraine is crucial, as it influences the decision to initiate dual antiplatelet therapy (107).

## 5 Antimigraine medications

Serotonin levels peak during migraine attacks leading to vasoconstriction and reducing blood flow, thereby alleviating the headache associated with migraine (11). Additionally, in ischemic conditions, when tissue blood supply is already compromised, serotonin’s vasoconstrictive effects are further amplified (11). This interaction highlights the critical role of serotonin not only in the neurobiology of migraines but also in how it can influence vascular responses under different physiological conditions.

The primary mechanism of action for many medications used to treat and prevent migraine attacks involves targeting serotonin receptors and the CGRP pathway. Drugs that act as serotonin receptor agonists help alleviate both the intensity and frequency of migraine symptoms by constricting the excessively dilated blood vessels in the dura mater, the outer membrane of the brain (114). Additionally, CGRP antibodies disrupt the activity of the CGRP peptide, also preventing the dilation of blood vessels and further ameliorating migraine symptoms (114). This dual strategy is instrumental in managing the vascular changes associated with migraines.

MRI studies have revealed that only extracranial vessels undergo dilation during migraines. Medications like sumatriptan precisely target these extracranial arteries inducing vasoconstriction without affecting intracranial vessels. While vasoconstriction is implicated in specific ischemic conditions such as vasospasm following subarachnoid hemorrhage and reversible cerebral vasoconstriction syndrome (RCVS), it is not the predominant cause of IS or TIA, which typically involves other factors such as atherosclerotic plaques, emboli, and chronic conditions like diabetes and hypertension (115). Both vasospasm and RCVS can present with a severe, sudden-onset (thunderclap) headache. RCVS may also involve focal neurological deficits. In these scenarios, the use of Triptans is contraindicated due to the potential for exacerbating vasoconstriction and thus worsening the patient’s condition (115).

Conversely, certain anti-migraine medications exhibit vasodilatory effects. For instance, beta-blockers such as propranolol, Non-steroidal anti-inflammatory drugs (NSAIDs) such as tolfenamic acid, and ergot derivatives such as nicergoline help alleviate migraine symptoms by relaxing blood vessels, which can reduce the pressure and pain during a migraine attack and offer alternative therapeutic options for patients with specific vascular concerns (114). However, the consumption of NSAIDs by migraineurs who experience at least 10 attacks per month may provoke more migraine attacks than it prevents and is also a risk factor for HS (116).

Interestingly, antiplatelet therapies such as aspirin and clopidogrel, used for the secondary prevention of non-cardioembolic TIA and IS, can also incidentally decrease migraine attacks (117, 118). Trabattoni et al. reported that P2Y12 antagonists, such as clopidogrel, significantly reduced the levels of tissue factor, reactive oxygen species, and microvesicles in the plasma of patients with PFO-MA (61). Additionally, increasing the dosage of aspirin from 81 mg to 300 or 325 mg may contribute to the secondary prevention of recurrent stroke and reduce migraine attacks (115). Valproic acid, beta-blockers, and OnabotulinumtoxinA are also deemed safe for migraine prevention in patients with a history of stroke (11, 115).

Calcium channel blockers (CCB) through their vasodilatory mechanism have been effective aids in migraine prevention. CCBs also have anti-ischemic properties through their interactions with GABA _A_ receptors in the brain. The resultant GABA released acts directly on vessels and indirectly as a neuromodulator through its role as an inhibitory neurotransmitter (114). Melatonin, another agent used for migraine prevention, also exerts an anti-ischemic effect by modulating GABA_A_ receptors on cerebral vasculature (114, 119).

Liao et al. in a 19-year cohort study revealed that acupuncture significantly lowered the occurrence of both IS and HS by 60% compared to a non-acupuncture group across all genders and age groups (120). The study also found that independent of acupuncture therapy, patients who received more medication for migraine attacks or prevention experienced fewer strokes. Interestingly, the study noted a higher prevalence of stroke in older migraineurs, contrasting with earlier research that suggested a greater impact of migraine on stroke risk in younger individuals (120). This underscores the intricate relationship between migraine management, vascular health, and therapeutic interventions across various medical modalities.

## 6 Conclusion

Our review elucidates the intricate and multifaceted relationships between migraine and stroke, revealing significant clinical implications for management and treatment. Migraine not only shares pathophysiological mechanisms with stroke but also enhances the predisposition to certain types of strokes. This underscores the necessity of understanding their interplay to manage these conditions effectively.

The categorization of IS in migraine patients, particularly migrainous infarction, requires meticulous attention to clinical and imaging criteria to ensure precise diagnosis.

MA significantly increases the risk of IS depending on attack frequency. However, its association with HS remains uncertain due to conflicting research findings. Factors like CSD, PFO, and conditions such as systemic lupus erythematosus and antiphospholipid syndrome contribute to this heightened IS risk. Further focused research is needed to clarify these associations and develop effective stroke prevention strategies for migraine patients.

Genetic predispositions contribute to an elevated risk of vascular pathologies such as cervical artery dissection and various syndromes like CADASIL and MELAS. This highlights the critical role of genetic research in clarifying the connections between migraine and stroke.

Endothelial dysfunction emerges as a pivotal mechanism linking migraine to stroke, characterized by impaired vascular reactivity and dysregulated inflammatory pathways. Biomarkers of this dysfunction, such as endothelial microparticles and inflammatory cytokines, offer valuable insights into the underlying pathophysiological processes.

A persistent PFO into adulthood poses significant risks for ischemic stroke and MA. While PFO closure reduces recurrent stroke risk, its impact on migraine management remains uncertain.

AF complicates both IS and MA through bidirectional associations. AF predisposes to stroke via cardioembolic mechanisms, while migraine-associated autonomic dysfunction can trigger AF. Variability in studies underscores the need for tailored management strategies, with AF treatment focusing on stroke prevention and ongoing research needed to clarify implications for migraine management.

Hormonal influences, especially fluctuations in estrogen levels, play a substantial role in the pathogenesis of migraine and its associated stroke risk, necessitating tailored therapeutic interventions for vulnerable populations such as women of childbearing age and postmenopausal women.

The relationship between migraine and structural brain changes, including WMLs and SBIs, is complex. WMLs are more prevalent in migraine sufferers, especially those with MA, potentially predisposing them to deep WMLs and posterior circulation SBIs. Longitudinal studies suggest WMLs may increase over time in women with migraines, but the evidence for SBIs is inconsistent. Endothelial dysfunction, indicated by markers like ADMA and SDMA, may contribute to these abnormalities. Meta-analyses present conflicting findings on migraine’s association with SBIs due to variations in study design and populations.

Accurate diagnosis of stroke mimics and chameleons in migraine patients is essential to prevent mismanagement and optimize outcomes. Utilizing clinical scoring systems and advanced imaging techniques can aid in distinguishing between these entities, facilitating prompt and appropriate treatment.

The medical management of migraine in the context of stroke risk requires a nuanced approach that considers both the efficacy of antimigraine medications and individual risk factors, underlying cerebrovascular pathology, and treatment goals. In addition to pharmacological interventions, non-medical therapies such as acupuncture have shown promise in reducing stroke risk among migraineurs.

Although there are few studies on predicting stroke or its recurrence in patients with migraine headaches, smoking and oral contraceptive use can elevate stroke risk in this population. Advising patients on appropriate contraception methods and smoking cessation can mitigate future stroke risk. Like the general population, individuals with migraines should undergo evaluation for modifiable risk factors such as blood pressure, blood glucose levels, low-density lipoprotein levels, healthy nutritional habits, and exercise. They should then be guided toward suitable medical therapies or lifestyle modifications.

In conclusion, the complex interplay between migraine and stroke involves a myriad of genetic, vascular, hormonal, and neurobiological factors, among others. A comprehensive grasp of these dynamics is vital for devising targeted therapeutic strategies and enhancing clinical outcomes. Ongoing research is essential to unravel further complexities of this relationship and identify novel intervention and prevention strategies.

## Author contributions

VR: Writing – original draft, Writing – review & editing. SO: Writing – original draft, Writing – review & editing. RB: Conceptualization, Supervision, Writing – original draft, Writing – review & editing.

## Glossary

IS: ischemic stroke
HS: hemorrhagic stroke
ICHD-3: International Classification of Headache Disorders, 3rd Edition
MA: migraine with aura
MO: migraine without aura
CSD: cortical spreading depression
PFO: patent foramen ovale
mRS: modified Rankin Scale
CAD: coronary artery disease
GWAS: genome-wide association studies
LAS: large-artery stroke
CE: cardioembolic stroke
ACE: angiotensin-converting enzyme
MTHFR: methylene tetrahydrofolate reductase
CADASIL: cerebral autosomal dominant arteriopathy with subcortical infarcts and leukoencephalopathy
HERNS: hereditary epitheliopathy, retinopathy, nephropathy, and stroke (HERNS)
MELAS: mitochondrial encephalomyopathy, lactic acidosis, stroke-like symptoms
FHM: Familial hemiplegic migraine
SD: spreading depression
TIA: transient ischemic attack
CVR: cerebral vascular reactivity
CGRP: calcitonin gene-related peptide
MMP: matrix metalloproteinase
EMP: endothelial microparticles
EPC: endothelial progenitor cell
hs-CRP: high-sensitivity C-reactive protein
NO: nitric oxide
HR: hazard ratio
CI: confidence interval
CHC: combined hormonal contraceptives
HRT: hormone replacement therapy
OR: odds ratio
POP: progestin-only pills
WML: white matter lesions
SBI: subclinical brain infarcts
ADMA: asymmetric dimethylarginine
SMDA: symmetric dimethylarginine
MUMS: migraine with unilateral motor symptoms
NIHSS: National Institutes of Health Stroke Scale
RCVS: reversible cerebral vasoconstriction syndrome
NSAIDs: non-steroidal anti-inflammatory drugs
CCB: Calcium channel blockers
